# Supplementary material for: Comparative analysis of web-based programs for single amino acid substitutions in proteins
Source: PLoS One. 2022 May 4;17(5):e0267084. doi: 10.1371/journal.pone.0267084 (PMC9067658; doi:10.1371/journal.pone.0267084)

**Figure S1**: Distribution of deleterious/destabilizing mutations predicted by all 15 tools for PARK7.


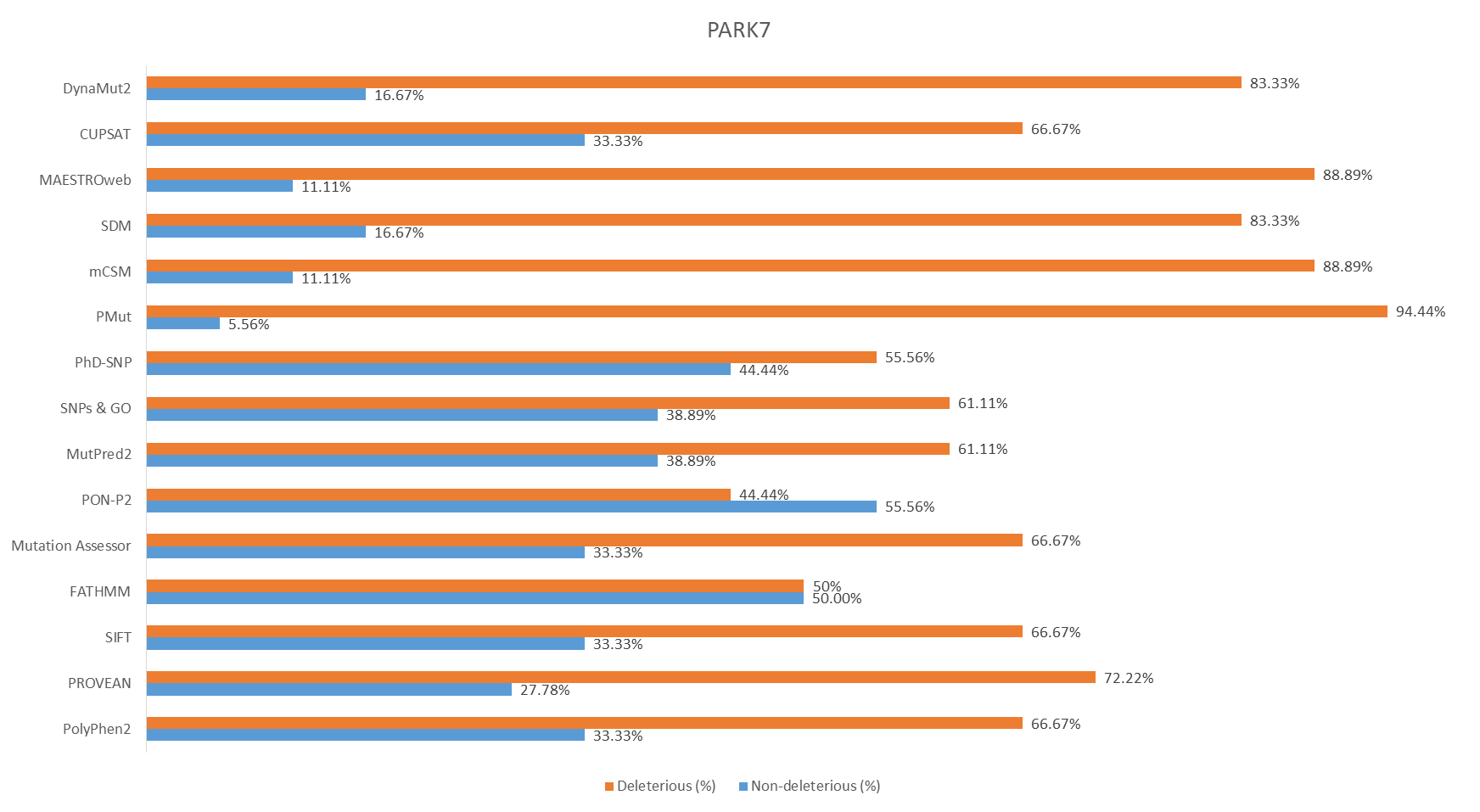

Supplement: S1 Fig — (DOCX) [file pone.0267084.s002.docx]
