# Supplementary material for: Comparative analysis of web-based programs for single amino acid substitutions in proteins
Source: PLoS One. 2022 May 4;17(5):e0267084. doi: 10.1371/journal.pone.0267084 (PMC9067658; doi:10.1371/journal.pone.0267084)
Supplement: S2 Fig — (DOCX) [file pone.0267084.s003.docx]

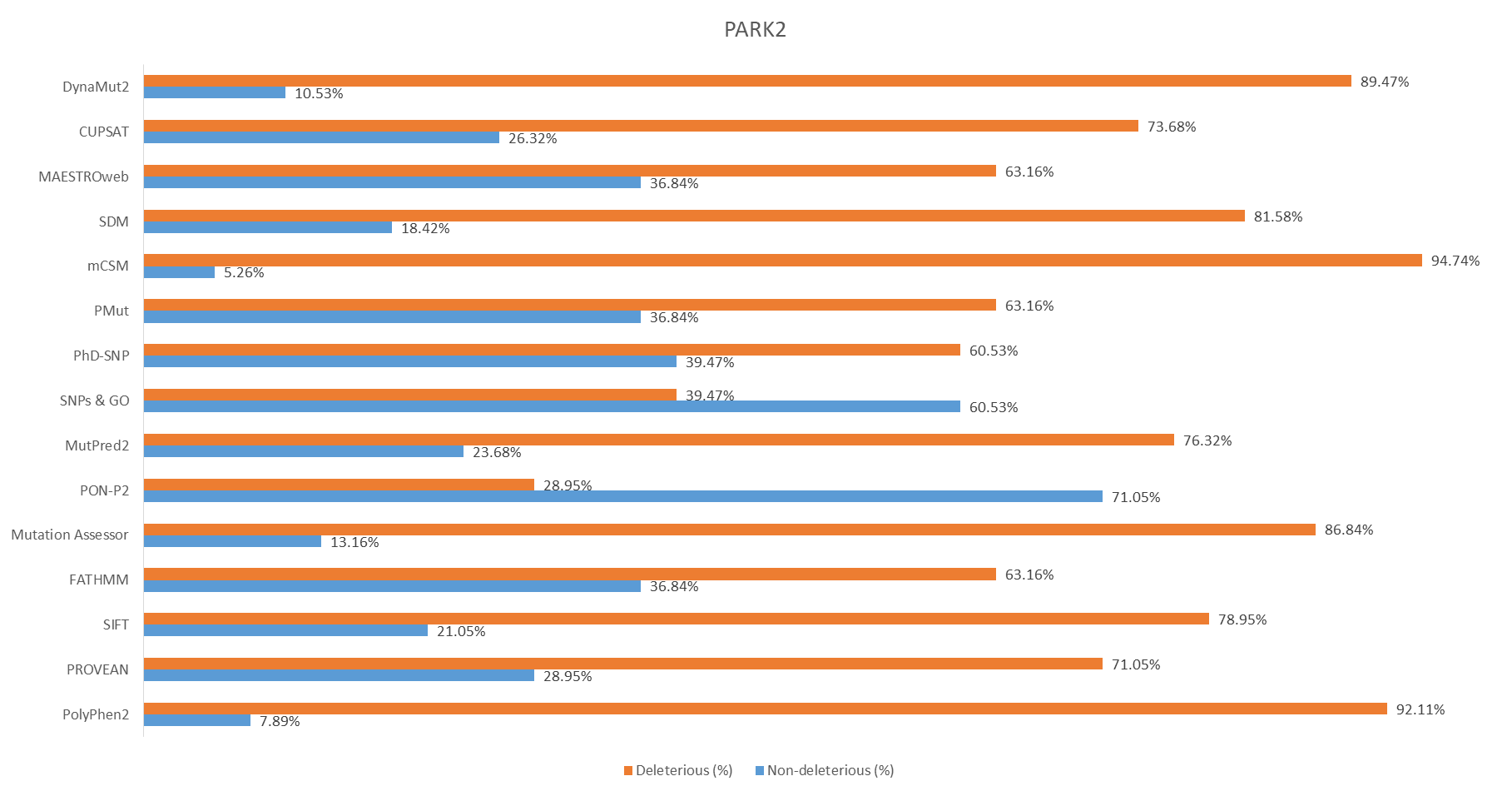


**Figure S2**: Distribution of deleterious/destabilizing mutations predicted by all 15 tools for PARK2.
